# Publisher Correction: Classification of magnetic order from electronic structure by using machine learning

**DOI:** 10.1038/s41598-023-40525-7

**Published:** 2023-08-21

**Authors:** Yerin Jang, Choong H. Kim, Ara Go

**Affiliations:** 1 Department of Physics, Chonnam National University, Gwangju, 61186 Korea; 2 Center for Correlated Electron Systems, Institute for Basic Science, Seoul, 08826 Korea; 3 Department of Physics and Astronomy, Seoul National University, Seoul, 08826 Korea

Correction to: *Scientific Reports* 10.1038/s41598-023-38863-7, published online 01 August 2023

The original version of this Article contained an error in Figure 4, where a single layer was distorted. The original Figure 4 and accompanying legend appear below.

**Figure 4:**
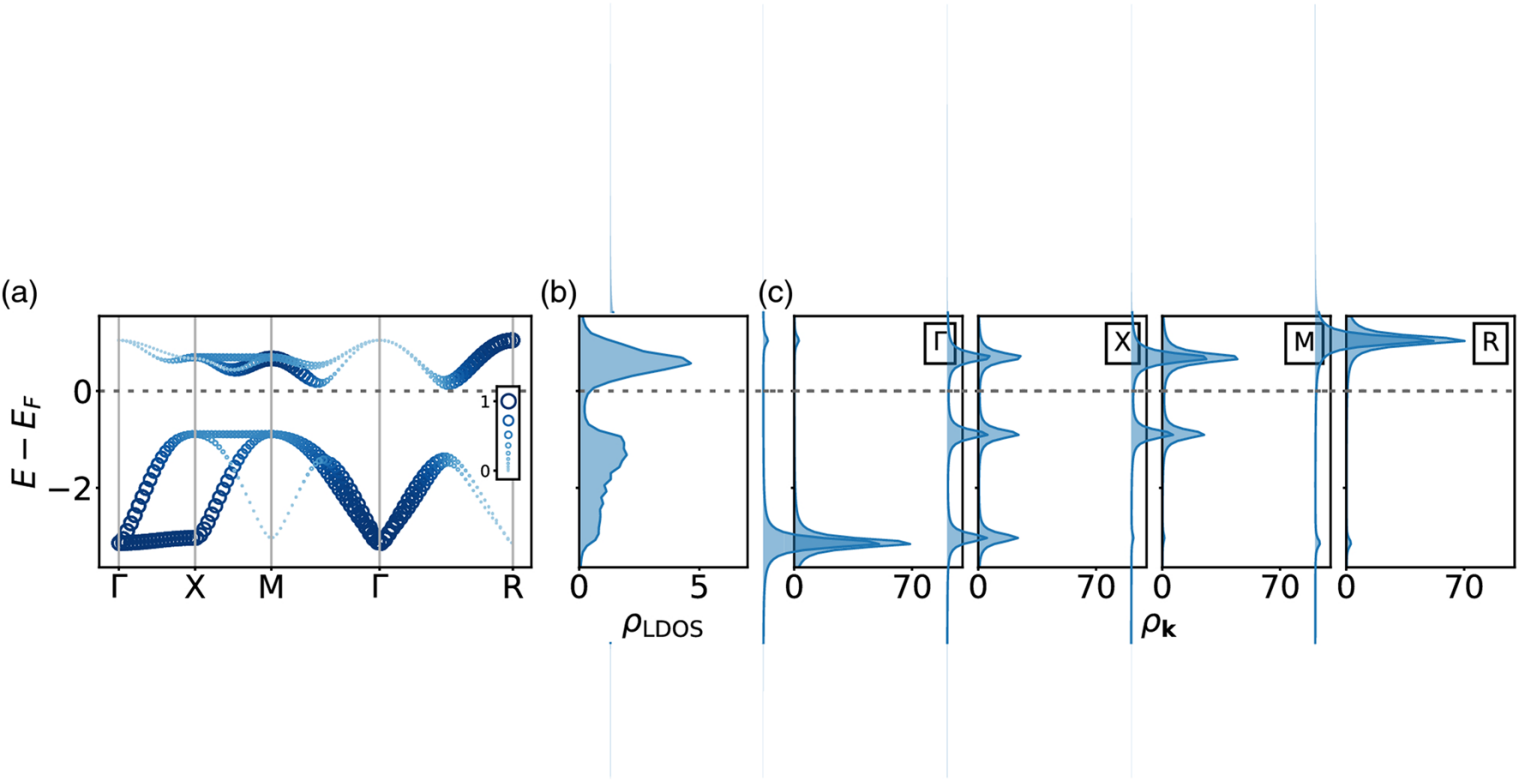
(**a**) Unfolded band structure to restore the original periodicity for G-type order with $N=3$ and $U=2$. Unlike the nonmagnetic bands whose weights are identical over all momenta, the unfolded bands are weighted ranging from 0 to 1. The color and the size of circle indicate the weights. (**b**) Corresponding local density of states ${\rho }_{\mathrm{LDOS}}\left(\omega \right)$ and (**c**) ${\text{k}}$-projected density of states ${\rho }_{\text{k}}(\omega )$ at high symmetry points with a broadening factor $\eta =0.1$.

The original Article has been corrected.

